# Obesity‐associated factors in psychiatric outpatients: A multicenter questionnaire survey

**DOI:** 10.1002/npr2.12465

**Published:** 2024-07-15

**Authors:** Hiroki Ishii, Hiroki Yamada, Ryotaro Sato, Wakaho Hayashi, Dan Nakamura, Shutaro Sugita, Taro Tazaki, Osamu Takashio, Atsuko Inamoto, Akira Iwanami

**Affiliations:** ^1^ Department of Psychiatry, Graduate School of Medicine Showa University Shinagawa‐ku, Tokyo Japan; ^2^ Department of Psychiatry, School of Medicine Showa University Shinagawa‐ku, Tokyo Japan; ^3^ Department of Psychiatry, East Hospital Showa University Hospital Shinagawa‐ku, Tokyo Japan; ^4^ Department of Psychiatry Showa University Northern Yokohama Hospital Tsuzuki‐ku, Yokohama Japan; ^5^ Shinrin Koen Mental Clinic Namekawa‐cho, Hiki‐gun, Saitama Japan; ^6^ Showa University Karasuyama Hospital Setagaya‐ku, Tokyo Japan; ^7^ Social, Genetic, and Developmental Psychiatry Centre Institute of Psychiatry, Psychology, and Neuroscience, King's College London London UK

**Keywords:** obesity, outpatient, pharmacotherapy, psychotropic drugs, side effects

## Abstract

The prevalence of obesity is increasing worldwide, resulting in various health issues such as hypertension, dyslipidemia, diabetes mellitus, heart disease, and a lower life expectancy. Importantly, several psychiatric disorders and the use of psychotropic medications have been linked to obesity, and the possible risk factors need further investigation. This study examined the prevalence of obesity and its associated factors using a self‐administered questionnaire. Participants were recruited from three outpatient clinics and individuals who met one or more of the ICD‐10 F0‐F9, G4 diagnoses were included. In total, 1384 participants completed the questionnaire about their lifestyle. Statistical analysis compared the demographic and clinical characteristics of the individuals who were obese (Body Mass Index: BMI ≥25) and those who were non‐obese (BMI <25). The results revealed that the factors associated with obesity in psychiatric outpatients were being male, prolonged treatment duration, eating out frequently, and use of both second‐ and first‐generation antipsychotics. The study emphasized the importance of closely monitoring BMI in individuals with multiple obesity‐related factors.

## Abstract 2

A survey of 1384 psychiatric patients identified factors related to BMI >25. Results showed that obesity was associated with gender, dining out frequency, hospitalizations, antipsychotic medication use, and treatment duration.

## Graphical abstract

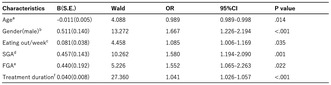

## 1 INTRODUCTION

Obesity is having excess body weight and fat in body fat tissue. Body Mass Index (BMI), which is calculated by dividing weight in kilograms by height in meters squared, defines obesity in most countries, including Japan. Internationally, BMI >30 is defined as obese, but The Japan Society for the Study of Obesity classifies body weight as “underweight (thin)” if BMI is <18.5, “normal weight” if it is between 18.5 and 25 and “obese” if BMI is 25 or more.

Obesity is a growing problem worldwide, particularly among young people and in developed countries. The prevalence of obesity has doubled in more than 70 countries since 1980 and is continuously increasing in most other countries.^1^ In 2015, a total of 107.7 million children and 603.7 million adults were obese worldwide.^1^ Obesity can lead to various health problems, including shortened life expectancy, type 2 diabetes, cardiovascular disease, certain cancers, kidney disease, obstructive sleep apnea, gout, osteoarthritis, and hepatobiliary system disease.^2^, ^3^

Importantly, obesity has been linked to various psychiatric disorders. For example, individuals with schizophrenia are known to be at high risk of premature mortality due to poor physical health, especially cardiovascular disease, diabetes, and obesity.^4^ Moreover, Bioque et al.^5^ suggested that obesity in schizophrenia may not only be a result of the chronic course and treatment of the disease but also occur early in its onset. Depression has also been associated with obesity. Milaneschi et al.^6^ pointed out a bidirectional relationship between depression and obesity. They suggested that decreased activity, disorganized eating, and poor self‐care resulting from depressed mood and low motivation may contribute to the higher rate of obesity in depression‐affected individuals.^6^ Furthermore, adults with bipolar disorder were 60% more likely to be obese than those without the condition.^7^ They had an increased risk of developing type 2 diabetes, cardiovascular disease, metabolic syndrome, cognitive impairment, and dementia.^8^ Developmental disorders such as autism spectrum disorder (ASD) and attention‐deficit/hyperactivity disorder (ADHD) are also associated with a high prevalence of obesity. The nationwide study conducted by Healy et al. found that children and teenagers with ASD had a higher risk of being overweight or obese in comparison with a non‐ASD population, with odds ratios of 1.48 and 1.49 for being overweight and obese, respectively.^9^ Similarly, studies have reported that the odds ratios for being obese in individuals with ADHD in comparison with non‐ADHD counterparts were 1.20 and 1.55 for children and adult populations, respectively.^10^

The use of psychotropic medications may mediate obesity significantly in individuals with psychiatric disorders. For instance, weight gain is commonly observed in patients taking antipsychotic drugs for schizophrenia.^11^ Correll et al.^12^ showed that metabolic syndrome is highly prevalent in patients with schizophrenia who were on second‐generation antipsychotics. Several risk factors for obesity were implicated in adolescents with schizophrenia who were on psychotropic medications, such as being male, presenting affective symptoms, early onset, and polypharmacy, including antidepressants and mood stabilizers.^5^ Another study with hospital inpatients with bipolar disorder found that the single use of atypical antipsychotics was associated with greater weight gain than typical antipsychotics or mood stabilizers.^13^ In their study, the obesity rate increased from 25% to 36% in the first 4 weeks of starting the treatment.^13^

Further, obesity and related health issues may worsen the prognosis of schizophrenia and other psychiatric disorders. Studies have shown that individuals with serious mental illnesses (SMI) such as schizophrenia, bipolar disorder, and major depressive disorder had a higher mortality rate and shorter life expectancy than those who were unaffected.^14^, ^15^, ^16^, ^17^, ^18^ The primary reason for this is a physical illness, especially cardiovascular disease (CVD).^19^, ^20^ Obesity and other metabolic abnormalities are significant risk factors for CVD,^21^ which are particularly common in patients with SMI.^14^, ^15^ Moreover, unhealthy lifestyle habits and psychotropic medications further increase the risk of CVD.^22^ Identifying factors that contribute to weight gain in clinical settings and implementing effective countermeasures are crucial in improving the quality of treatment and life expectancy in people with psychiatric disorders.

This study, therefore, examined the demographic and clinical factors associated with obesity in outpatients with psychiatric disorders using a self‐administered questionnaire at three different facilities in Japan.

## 2 METHODS

### 2.1 Participants

Participants were recruited from outpatients who visited Showa University Karasuyama Hospital (a psychiatric hospital), Showa University Northern Yokohama Hospital (a general hospital with a psychiatric department), and Shinrin‐koen Mental Clinic (a psychiatric clinic) between January and April 2020. We included those who received one or more diagnoses of International Classification of Diseases 10th Revision (ICD‐10) F0 to F9 (F0: organic mental disorders including symptomatic, F1: mental and behavioral disorders due to psychoactive substance use, F2: schizophrenia, schizotypal disorder, and delusional disorder, F3: mood [affective] disorder, F4: neurotic disorder, stress‐related disorder, and somatoform disorder, F5: physiological disorders and behavioral syndromes related to physical factors, F6: personality and behavioral disorders in adults, F7: intellectual disability, F8: disorders of psychological development, F9: behavioral and emotional disorders that usually occur in childhood and adolescence) and G4 (interictal and seizure disorders). The exclusion criteria were those who did not consent to the study. In total, 1721 participants were recruited, and 1384 individuals (mean age, 48.2 years; 650 men) were eligible and included in the study.

### 2.2 Questionnaire

Table 1 shows the self‐administered questionnaire used in the study. It consists of open‐ended questions about age, sex, treatment duration, height, weight, living situation, frequency of eating out, exercise habits, whether one feels any stress or not (stress), and alcohol and smoking use.

**TABLE 1:** Questionnaires distributed.

Age	Years old
Sex	M/F
Height	cm
Body weight	kg
How many years has it been since you first became ill?	Years
How many years has it been since you first started treatment?	Years
Do you live alone?	Yes/No
How many days per week do you exercise?	Days
How many days per week do you eat out?	Days
Are you currently feeling stressed?	Yes/No
Do you drink alcohol?	Yes/No
Do you smoke?	Yes/No

### 2.3 Procedure

The questionnaire was administered only once, and all participants completed it on the spot when they visited one of the facilities involved. The medical records were used to collect information about the participants' illness and treatment duration, medication history, and the type and amount of medication taken on the day of the questionnaire administration.

### 2.4 Statistical analysis

A descriptive analysis was performed to summarize the characteristics of the participants. We then divided the participants into two groups: obese (BMI ≥25) and non‐obese (BMI <25) groups, and performed a between‐group analysis of the study variables (age, gender, duration of treatment, BMI, ICD‐10 diagnosis, stress, alcohol consumption, smoking, number of days/week of eating out, and number of days/week of exercise, and medications). For those who had multiple diagnoses, a primary diagnosis was adopted. The chi‐squared or Fisher's exact probability test was used for the nominal variables, and Mann–Whitney's U test was used for the continuous variables. Logistic regression analysis was used to examine the correlations between different factors such as age, gender, treatment duration, frequency of eating out, types of medication prescribed (First‐Generation Antipsychotics [FGAs], Second‐Generation Antipsychotics [SGAs], antiparkinsonian drugs, new and conventional antidepressants, benzodiazepine [BZD]) anxiolytic and sleep medications, mood stabilizers, ADHD and antidementia medications and the likelihood of obesity (BMI ≥25) among individuals without any missing data (*n* = 1284). The significance level was set at 0.05, and Microsoft Excel 2019 (Microsoft Corp., Redmond, WA) and SPSS (version 25.0; IBM Corp., Armonk, NY) were used for all statistical analyses.

### 2.5 Classification of psychotropic drugs

We categorized risperidone, olanzapine, quetiapine, perospirone, aripiprazole, paliperidone, blonanserin, brexpiprazole, and lurasidone (all antipsychotics launched in Japan after 1996) as SGAs and all other antipsychotics as FGAs. Fluvoxamine, paroxetine, milnacipran, duloxetine, mirtazapine, escitalopram, venlafaxine, and vortioxetine (all antidepressants launched in Japan after 1999) were categorized as newer antidepressants, whereas all other antidepressants were classified as conventional. Non‐benzodiazepines with GABAergic receptor agonist properties were included in the benzodiazepines. Mood stabilizers included lithium carbonate, valproate, carbamazepine, lamotrigine, and quetiapine extended‐release tablets. Quetiapine extended‐release tablets were included in the mood stabilizers in this study because their pharmacokinetics and maximum dosage differ from those of fast‐release tablets, and they are only indicated for bipolar disorder in Japan. None of the participants were using clozapine.

## 3 RESULTS

### 3.1 Demographic and clinical characteristics of the participants

In total, 1721 participants were recruited, and 1384 individuals were eligible and included in the study. The average age of participants was 48.2 (Standard deviation [SD], 17.0) years old, with the average disease and treatment durations of 14.3 (SD, 11.2) and 12.1 (SD, 10.1) years, respectively. The sex proportion was 47.0% (650 individuals) male to 53.0% (734 individuals) female, showing a slight female predominance. The average height, weight, and BMI were 163.1 (SD, 9.1) cm, 64.2 (SD, 16.1) kg, and 24.0 (SD, 5.1), respectively. The common diagnostic groups were F3, F2, and F4, for which 408 (29.5%), 337 (24.3%), and 211 (15.2%) individuals met one of the diagnostic criteria in each group, respectively. All F5 patients (*n* = 20, 1.4%) had eating disorders. The rate of obesity was high as 463 (33.5%) participants had a BMI ≥25, whereas 921 (66.5%) of them had a BMI <25 (Table 2).

**TABLE 2:** Demographic and clinical characteristics of participants.

Characteristics	Overall (*n* = 1384)	Obesity (BMI ≥25)		OR	*p* Value
		Yes (*n* = 463)	No (*n* = 921)		
Mean age (SD), years	48.2 (17.0)	47.5 (13.8)	48.5 (18.4)		
Male, *n* (%)	650 (47.0)	255 (55.1)	395 (42.9)	1.63	<0.001
Mean duration of illness (SD), years^a^	14.3 (11.2)	17.1 (11.1)	13.0 (11.2)		<0.001
Mean duration of treatment (SD), years^b^	12.1 (10.1)	15.2 (10.6)	10.7 (9.6)		<0.001
Mean stature (SD), cm^c^	163.1 (9.1)	164.5 (9.0)	162.4 (9.1)		<0.001
Mean body weight (SD), kg^d^	64.2 (16.1)	79.4 (13.5)	56.0 (10.6)		<0.001
Mean BMI (SD)^e^	24.0 (5.1)	29.3 (3.9)	21.1 (5.9)		0.012
Diagnosis (ICD‐10)					
F0, *n* (%)	44 (3.2)	7 (1.5)	37 (4.0)	0.36	0.012
F1, *n* (%)	27 (2.0)	10 (2.2)	17 (1.8)	1.17	
F2, *n* (%)	337 (24.3)	152 (32.8)	186 (20.1)	1.94	<0.001
F3, *n* (%)	408 (29.5)	126 (27.2)	282 (30.6)	0.84	
F4, *n* (%)	211 (15.2)	55 (11.9)	156 (16.9)	0.66	0.013
F5, *n* (%)	20 (1.4)	4 (0.9)	16 (1.7)	0.49	
F6, *n* (%)	19 (1.4)	6 (1.3)	13 (1.4)	0.91	
F7, *n* (%)	18 (1.3)	7 (1.5)	11 (1.2)	1.26	
F8, *n* (%)	88 (6.4)	31 (6.7)	57 (6.2)	1.08	
F9, *n* (%)	206 (14.9)	62 (13.4)	144 (15.6)	0.83	
G4, *n* (%)	6 (0.4)	3 (0.6)	3 (0.3)	1.99	

*Note*: Data are presented as numbers (percentage), mean (standard deviation). For nominal variables, we used the *χ* ^2^ test, and for G4, Fisher's exact probability test was used. For continuous variables, we used Mann–Whitney's U test. Participants without missing values (a, *n* = 1320; b, *n* = 1334; c, *n* = 1340; d, *n* = 1326; e, *n* = 1324).

Abbreviations: OR, odds ratio; BMI = body mass index.

### 3.2 Between‐group comparison of backgrounds

Table 2 compares obese (BMI ≥25) and non‐obese (BMI <25) groups on various background characteristics. The average height and weight of the obese group were 164.5 (SD, 9.0) cm and 79.4 (SD, 13.5) kg, which were significantly higher than those of the non‐obese group, which were 162.4 (SD, 9.1) cm and 56.0 (SD, 10.6) kg (*p* < 0.001). In the obese group, the proportion of males, which was 55.1% (255 individuals), was significantly higher than that of the non‐obese group, which was 42.9% (395 individuals). No difference was found in age, in which the average age of individuals in the obese and non‐obese groups was 47.5 (SD, 13.8) and 48.5 (SD, 18.4) years old, respectively. Participants in the obese group had a longer duration of disease and treatment than those in the non‐obese group, with an average of 17.1 (SD, 11.1) and 15.2 (SD, 10.6) years, respectively. The non‐obese group had an average of 13.0 (SD, 11.2) years for disease and 10.7 (SD, 9.6) years for treatment duration. The proportions of participants who met F0, F2, and F4 diagnoses showed significant between‐group differences. The proportion of individuals with F0 diagnoses in the obese group was 1.5%, which was significantly lower than 4.0% in the non‐obese group (*p* = 0.012). Similarly, the proportion of participants with F4 diagnoses in the obese group was 11.9%, which was significantly lower than 16.9% in the non‐obese group (*p* < 0.001). In contrast, the proportion of individuals with F2 diagnoses in the obese group was 32.8%, which was significantly higher than 20.1% in the non‐obese groups (*p* = 0.013).

### 3.3 Between‐group comparison of lifestyle

Table 3 compares the lifestyle variables between the obese and non‐obese groups. Significant between‐group differences were found in the habit and frequency of eating out and smoking. Specifically, 68.5% of individuals in the obese group had the habit of eating out, which was significantly higher than 59.7% of the non‐obese group (*p* = 0.002). Similarly, individuals in the obese group ate out more frequently than their non‐obese counterparts; the average days of eating out were 2.5 (SD, 1.9) and 2.3 (SD, 1.8) per week in the former and latter groups, respectively (*p* = 0.002). While 24.4% of individuals smoked regularly in the obese group, significantly fewer participants, 17.7%, in the non‐obese group smoked (*p* = 0.003). Interestingly, the proportion of individuals living alone, the habit and frequency of exercise, stress awareness, and drinking habits did not differ significantly between the obese and non‐obese groups.

**TABLE 3:** Lifestyle factors between the obese and non‐obese groups.

Characteristics	Overall (*n* = 1384)	Obesity (BMI ≥25)		OR	*p* value
		Yes (*n* = 463)	No (*n* = 921)		
Living alone, *n* (%)	297 (20.4)	109 (23.5)	188 (20.4)	1.20	
Habit of eating out, *n* (%)	867 (62.6)	317 (68.5)	550 (59.7)	1.46	0.002
Mean frequency of eating out/week (SD)	2.3 (1.8)	2.5 (1.9)	2.3 (1.8)		0.002
Habit of exercise, *n* (%)	773 (55.8)	260 (56.2)	513 (55.7)	1.01	
Mean frequency of exercise/week (SD)	3.1 (2.0)	3.1 (2.1)	3.2 (2.2)		
Feeling stressed, *n* (%)	962 (69.5)	325 (70.2)	637 (69.2)	1.04	
Alcohol intake	334 (24.1)	125 (27.0)	209 (22.7)	1.25	
Smoking	276 (19.9)	113 (24.4)	163 (17.7)	1.50	0.003

*Note*: Data are presented as numbers (percentage), mean (standard deviation). For nominal variables, we used the *χ* ^2^ test, and for G4, Fisher's exact probability test was used. For continuous variables, we used Mann–Whitney's U test.

Abbreviations: BMI, body mass index; OR, odds ratio.

### 3.4 Between‐group comparison of psychotropic prescription

Table 4 compares the prescription rates of psychotropic medications between the obese and non‐obese groups. Overall, the proportion of individuals who were prescribed any psychotropic medications in the obese group was 95.5%, which was significantly higher than 92.6% of the non‐obese group (*p* = 0.042). In terms of antipsychotics, the obese group had significantly higher rates not only of total prescription but also of SGA and FGA prescriptions than the non‐obese group (*p* < 0.001). Specifically, in the obese group, 61.3%, 54.2%, and 20.5% of the participants were prescribed antipsychotics, SGA, and FGA, respectively, whereas in the non‐obese group, these proportions were 42.9%, 38.4%, and 10.5%. The overall prescription rate of antidepressants was similar between the two groups (43.2% in the obese and 42.6% in the non‐obese group), with no significant between‐group differences in the types of antidepressants prescribed. However, the prescription rate of BZDs was significantly higher in the obese (60.7%) than in the non‐obese group (55.5%) (*p* = 0.002). Similarly, the obese group had higher prescription rates of mood stabilizers (27.3%) and antiparkinsonian drugs (20.7%) than the non‐obese group (18.9% and 11.6%, respectively; both *p* < 0.001). No between‐group difference was found in the prescription rates of ADHD and anti‐dementia medications.

**TABLE 4:** Psychotropics prescription rates between the obese and non‐obese groups.

Psychotropics	Overall (*n* = 1384)	Obesity (BMI ≥25)		OR	*p* value
		Yes (*n* = 463)	No (*n* = 921)		
Any psychotropic drugs, *n* (%)	1295 (93.4)	442 (95.5)	853 (92.6)	1.68	0.042
Any antipsychotics, *n* (%)	679 (49.1)	284 (61.3)	395 (42.9)	2.11	<0.001
SGA, *n* (%)	605 (43.7)	251 (54.2)	354 (38.4)	1.89	<0.001
FGA, *n* (%)	192 (13.9)	95 (20.5)	97 (10.5)	2.19	<0.001
Any antidepressants, *n* (%)	592 (42.8)	200 (43.2)	392 (42.6)	1.02	
Newer antidepressants, *n* (%)	511 (36.9)	167 (36.1)	344 (37.4)	0.94	
Conventional antidepressannts, *n* (%)	152 (11.0)	56 (12.1)	96 (10.4)	1.18	
Any BZDs, *n* (%)	792 (57.2)	281 (60.7)	511 (55.5)	1.23	
BZDs hypnotics, *n* (%)	540 (39.0)	207 (44.7)	333 (36.2)	1.42	0.002
BZDs anxiolytics, *n* (%)	523 (37.8)	184 (39.7)	339 (36.8)	1.13	
Mood stabilizers, *n* (%)	310 (22.4)	158 (27.3)	152 (18.9)	1.52	0.001
ADHD Medications, *n* (%)	199 (14.4)	82 (14.2)	117 (14.5)	0.93	
Antidementia drugs, *n* (%)	25 (1.8)	14 (2.4)	11 (1.4)	0.20	
Antiparkinsonian drugs, *n* (%)	203 (14.7)	96 (20.7)	107 (11.6)	1.98	<0.001

*Note*: Data are presented as numbers (percentage). For nominal variables, we used the *χ* ^2^ test. For continuous variables, we used Mann–Whitney's U test.

Abbreviations: ADHD, attention‐deficit/hyperactivity disorder; BMI, body mass index; BZD, benzodiazepine; FGA, first‐generation antipsychotics; OR, odds ratio; SGA, second‐generation antipsychotics.

### 3.5 Factors associated with obesity (BMI ≥25)

A logistic regression analysis (Table 5) showed that being male (OR: 1.667, 95% CI: 1.226–2.194, *p* < 0.001), higher frequency of eating out (OR: 1.085, 95% CI: 1.006–1.169, *p* = 0.035), SGA (OR: 1.080, 95% CI: 1.006–1.169, *p* = 0.035) and FGA prescription (OR: 1.552, 95% CI: 1.065–2.263, *p* = 0.022), and longer treatment duration (OR: 1.041, 95% CI: 1.026–1.057, *p* < 0.001) were significantly associated with obesity. In addition, a significant negative association was found between age and having a BMI ≥25 (OR: 0.989, 95% CI: 0.989–0.998, *p* = 0.014).

**TABLE 5:** Odds ratios for obesity (BMI ≥25) by participants' characteristics (participants without missing values: *n* = 1284).

Characteristics	B (SE)	Wald	OR	95%CI	*p* Value
Age^a^	−0.011 (0.005)	4.088	0.989	0.989–0.998	0.014
Gender (male)^b^	0.511 (0.140)	13.272	1.667	1.226–2.194	<0.001
Eating out/week^c^	0.081 (0.038)	4.458	1.085	1.006–1.169	0.035
SGA^d^	0.457 (0.143)	10.262	1.580	1.194–2.090	0.001
FGA^e^	0.440 (0.192)	5.226	1.552	1.065–2.263	0.022
Treatment duration^f^	0.040 (0.008)	27.360	1.041	1.026–1.057	<0.001

*Note*: Multiple logistic regression analysis forward stepwise entry method.

Abbreviations: CI, confidence interval; FGA, first‐generation antipsychotics; OR, odds ratio; SE, standard error; SGA, second‐generation antipsychotics.

^a^ Value per 1 year increase.

^b^ Female coded 0, male coded 1.

^c^ Value per 1 day/week increase.

^d^ Not used SGA coded 0, used SGA coded 1.

^e^ Not used FGA coded 0, used FGA coded 1.

^f^ Value per 1 year increase.

## 4 DISCUSSION

This study aimed to investigate the factors contributing to obesity among psychiatric outpatients in Japan. By comparing the obese group (BMI ≥25) and non‐obese group (BMI <25), the researchers found that the obese group had a higher proportion of men, longer duration of disease and treatment, higher smoking rates, and a significantly higher usage of SGA, FGA, BZD sleeping pills, mood stabilizers, and antiparkinsonian drugs than the non‐obese group. After multivariate analysis, it was found that male gender, treatment duration, eating out frequency, and use of SGA and FGA were significantly associated with obesity (BMI ≥25).

Obesity proneness in men found in our study has been reported in the general population. Lifestyle and daily habits can be considered as one of the strong influences on our study participants as they were not hospitalized. According to the National Health and Nutrition Survey Report by the Ministry of Health, Labour and Welfare, the percentage of men and women living with obesity in Japan aged 20 and above were 33.0% and 22.3%, respectively.^23^ A community‐based survey with 5425 residents aged between 20 and 64 years old in Kobe city also found that the prevalence rate of obesity in men (27.2%) was more than double that of women (10.6%).^24^

Men in full‐time work have more abdominal fat than their female counterparts because they spend the majority of their time sitting at a desk^25^ and their overtime work and late dinner times,^26^ together with high alcohol consumption^4^ and heavy smoking,^27^ may contribute to their obesity. Our male participants had a higher rate of smoking than their female counterparts, which may partly explain the lifestyle in men affecting obesity, as heavy smoking has been linked to obesity. Further studies, including employment status, working hours (time sitting at a desk), and between‐sex comparisons, are required to examine the association between male gender and obesity.

Although there is no existing literature on the frequency of eating out and obesity in individuals with psychiatric disorders, the Japanese National Health and Nutrition Examination Survey reported that the number of individuals living with obesity (BMI ≥25) increased when they ate out and had lunch boxes twice a week or more.^28^ A study of 35 084 US adults found that those with more than two meals prepared daily outside the home had a higher risk of all‐cause mortality.^29^ This was compared to those who had less than one meal prepared outside the home per week after adjusting for diet, lifestyle factors, and BMI.^29^ Eating habits that do not involve cooking at home, such as eating out or packing lunches, may lead to nutritional imbalances, increasing the risks of obesity and CVD in general populations.

It is likely that this finding with the general population also applies to psychiatric outpatients, and they may have unhealthy dietary habits. For example, Dipasquale et al. found that individuals with schizophrenia tended to have an unhealthy diet that was high in saturated fat and low in fiber and fruit.^30^ Calkin et al.^7^ speculated that weight gain during the acute phase of bipolar disorder was probably due to lack of exercise, difficulty maintaining a healthy diet, and exposure to medications. An unbalanced diet and its link to obesity has also been reported in autistic people and those with ADHD.^31^, ^32^, ^33^, ^34^, ^35^, ^36^, ^37^, ^38^, ^39^, ^40^, ^41^, ^42^, ^43^ For instance, children with ASD have a reduced variety of food they consume,^31^, ^32^, ^33^, ^34^, ^35^, ^36^ which may contribute to their obesity.^37^ Moreover, depressive symptoms in individuals with ADHD and obesity predicted their munching behaviors.^37^ Dakanalis et al. reported that overeating/obesity and unhealthy eating behaviors (e.g., fast food consumption) were associated with emotional eating, which has been linked to depressive symptoms.^44^ While it is possible that poor dietary habits and unbalanced nutrition from eating out can cause obesity, further research is necessary to determine any obesity‐related factors associated with dining out. These factors may include the nutritional content of meals consumed while eating out and the timing and frequency of meals among individuals with psychiatric disorders.

Antipsychotic drugs can have various effects on the body, including weight gain.^3^, ^14^, ^45^, ^46^, ^47^, ^48^, ^49^, ^50^, ^51^ Our result that SGA and FGA prescription rates were significantly associated with obesity is consistent with previous findings. Allison et al. reported that antipsychotic‐treated patients who were on olanzapine and clozapine gained a significant amount of weight after 10 weeks of treatment, and various SGAs and several FGAs (e.g., thioridazine and haloperidol had been linked to weight gain).^45^ According to a meta‐analysis by Wu et al.,^46^ amisulpride, aripiprazole, brexpiprazole, cariprazine, haloperidol, lumateperone, and lurasidone caused mild weight gain in patients with acute exacerbation as compared to placebo. However, all the other medications resulted in more significant weight gain for most drugs, and dose–response curves indicated an initial dose‐related increase in weight, which plateaued at higher doses.^46^ A network meta‐analysis conducted by Pillinger et al.^47^ found that clozapine and olanzapine had a high risk of weight gain in patients with schizophrenia. In a review by Hasnain et al.,^48^ clozapine and olanzapine showed a relatively high risk of weight gain. In contrast, amisulpride, aripiprazole, and ziprasidone had a lower risk, and the other second‐generation antipsychotics showed moderate risk. Tek et al.,^49^ in their meta‐analysis, reported significant weight gain and BMI increase in association with antipsychotic use, including SGA and FGA, in patients with first‐episode psychosis, except for ziprasidone. According to a review by Correll et al., Individuals who were prescribed clozapine, olanzapine, and low‐potency FGA were found to have a higher risk of developing obesity.^14^ Pharmacologically, it has been shown that blocking the 5‐HT2c receptor can increase food intake while blocking the adrenergic β3 receptors found in adipose tissue can hinder the conversion of fat to energy and lead to weight gain.^50^ Additionally, blocking the histamine H1 receptor may increase hunger, and blocking the muscarinic M3 receptor may impact glucose regulation, resulting in weight gain.^50^ Further, the specific receptor affinity of SGA to histamine H1 receptors and the balance of dopamine D2 receptor and serotonin 5‐HT2 receptor binding affinity may play a role in weight gain.^51^ However, the effects of antipsychotic medications on weight gain may vary depending on the drug^47^ and individual factors such as genetics and metabolism.^3^ More research is needed to conclude how each antipsychotic affects weight gain in people with psychiatric disorders and mediating risk factors.

In the obese group, there was a higher use of antiparkinsonian medications, likely due to the increased use of antipsychotic medications in combination. Although univariate analysis indicated a higher use of antiparkinsonian medications in the obese group, multivariate analysis showed no direct link between obesity and the use of antiparkinsonian medications. Therefore, it was suggested that the influence of antipsychotic medications on obesity was likely more significant.

Contrary to our results, the previous literature mostly points out the risk of obesity in association with the use of antidepressants. For example, a cohort study of 20 751 Australian adults showed that antidepressant users were more likely to gain 3% or more of body weight per year, regardless of the type of antidepressants, frequency, and duration of the use.^52^ Salvi et al. used the Psychoactive Drug Screening Program (PDSP) Ki database to examine the affinity of antidepressants to receptors linked to weight change.^53^ They found that the H1 receptor was the strongest predictor of antidepressant‐induced weight gain. Similarly, Alonso‐Pedrero et al.^54^ reported in their review of 27 cohort studies that individuals using antidepressants showed approximately 5% weight gain. However, as some studies have highlighted, antidepressant‐induced weight gain may vary between drugs and occur more slowly or mildly than SGAs.^14^, ^18^, ^50^, ^55^ Carvalho et al.^56^ found that antidepressants, primarily mirtazapine and paroxetine, and tricyclic antidepressants, were associated with weight gain. In contrast, other selective serotonin reuptake inhibitors (SSRIs) showed a lower risk of weight gain during acute treatment.^56^ Lee et al.^57^ noted that SSRI use may be associated with weight loss during acute treatment and the risk of long‐term weight gain. In a review by Mazereel et al.,^18^ it was noted that the weight gain effects of antidepressants are more modest than those of antipsychotics. Similarly, the risk of obesity in mood stabilizer use seems to vary depending on the type. For instance, Grootens et al.^58^ found that valproic acid increased the risk of weight gain while carbamazepine was associated with a lower risk of it and lamotrigine with weight loss. A meta‐analysis by Gomes‐da‐Costa et al.^59^ concluded that weight gain from lithium was insignificant compared with placebo. There was no strong evidence of a direct link between obesity and other categories of psychotropic drugs. Other confounding factors may have a greater influence on psychotropic medications other than SGA and FGA, which were significantly different in the univariate analysis of this study.

Our analysis suggests that individuals receiving antipsychotic medications as psychiatric outpatients are at greater risk of developing obesity compared to those receiving antidepressants, mood stabilizers, and other categories of psychotropics. However, nonsignificant results may be due to the analysis not specifying the type and duration of the drug used, and further longitudinal studies are necessary to clarify the impact of psychotropic drugs, apart from antipsychotics, on obesity. Furthermore, it is currently uncertain whether psychiatric disorders themselves, the severity of psychiatric disorders, cause obesity, or whether medication is the root cause of this outcome. Further research is required to establish a clear relationship between psychotropic drugs, psychiatric disorders, and obesity.

A significant association between obesity and treatment duration was found in our study, which is in line with previous findings. In the meta‐analysis done by Mitchell et al.,^60^ the incidence of metabolic syndrome in patients with the first episode of schizophrenia was low, suggesting the risk of developing metabolic syndrome may increase with a longer duration of illness. Adachi et al.^61^ found a significant association between the period in which patients with bipolar disorder were in medical care and the use of multiple psychotropic medications. Tirupati et al.^62^ reported that 59% of patients in a psychiatric rehabilitation program had obesity. In comparison, 68% had metabolic syndrome, and patients who were on multiple medications had a higher frequency of metabolic syndrome than those who were not obese.^62^ Similarly, Hjorth et al.^63^ found that patients with severe psychiatric disorders, including schizophrenia, who were given multiple antipsychotic medications had a significantly larger waist circumference than those who were on a single medication. The potential role of polypharmacy due to prolonged treatment in increasing the risk of drug‐related obesity should be examined in a variety of clinical settings and pharmacological management.

To our knowledge, no study has shown a significant association between age and weight loss in patients with mental illnesses. In general populations, weight loss occurs over 65 due to muscle mass loss and bone demineralization.^64^, ^65^ Moreover, as people age, their dietary intake naturally decreases.^66^ A study conducted by Andres et al. in the United States revealed that the number of individuals who were obese (BMI of 30 or higher) increased until the age of 60 and then decreased at a later age.^67^ It is also possible that elderly patients experienced weight loss due to physical illnesses like cancer and diabetes mellitus, as discussed by Weverling‐Rijnsburger et al.^68^ They have emphasized the increased risk of developing chronic physical conditions in the elderly, which may lead to weight loss. Although the present study did not investigate physical comorbidities, the results might reflect these general changes.

Our study identified factors associated with obesity in outpatient psychiatric patients. Hjorthøj et al.^69^ reported that the life expectancy of schizophrenic patients is 15 years shorter than that of the general population. To support the long‐term health of psychiatric patients, it is important to examine potential risk factors of obesity and provide lifestyle guidance and appropriate medication management at outpatient practice.

Several limitations need to be addressed in this study. First, we cannot establish any causal relationships because the research is cross‐sectional. Second, not all lifestyle factors were examined. Future studies should investigate more variables such as the frequency and regularity of meals, hours of sitting at a desk at work, and history of smoking or drinking. Third, the self‐reported questionnaire may not accurately reflect real lifestyle and habits. Fourth, the relationship between obesity and disease has not been examined. Fifth, we could not include the study of abortive medications in this research. Sixth, we did not have the opportunity to assess the condition (including body type) before beginning treatment. Lastly, we have not studied the dose‐dependent effects of drugs on obesity disorder medication or conducted dosage‐specific analysis, which limits our discussion.

## 5 CONCLUSION

The present study found that male gender, longer treatment duration, frequent eating out, the use of SGA or FGA medications, and younger age may be linked to obesity in psychiatric outpatients. It highlights the importance of understanding risk factors associated with obesity in individuals with psychiatric disorders, and future studies need to compare medicated and non‐medicated participants with various disorders and lifestyle factors.

## AUTHOR CONTRIBUTIONS

Hiroki Ishii designed the study, wrote the protocol, collected data, performed statistical analysis, and wrote the manuscript. Hiroki Yamada and Osamu Takashio wrote the protocol, collected data, performed statistical analysis, and verified the manuscript. Ryotaro Satou, Shutarou Sugita, Taro Tasaki, and Dan Nakamura collected data and verified the manuscript. Wakaho Hayashi performed statistical analysis, verified the manuscript, and proofread the English text. Atsuko Inamoto and Akira Iwanami made critical revisions to the manuscript for important intellectual content. All authors have contributed to the manuscript and approved this submission.

## FUNDING INFORMATION

The authors did not receive any specific funding for this work.

## CONFLICT OF INTEREST STATEMENT

HI declares no conflict of interest. HY has received advisory panel payments from Yoshitomi Yakuhin and speaker's honoraria from Astellas Pharmaceutical, Eisai, Janssen Pharmaceuticals, Lundbeck Japan, Meiji‐Seika Pharma, Kyowa Pharmaceutical, Mochida Pharmaceutical, Otsuka Pharmaceutical, Sumitomo Pharma, Viatris Pharmaceutical, and Yoshitomi Yakuhin within the past 3 years. RS and WH declare no conflict of interest. DN has received speaker's honoraria from Janssen Pharmaceuticals, Meiji‐Seika Pharma, Otsuka Pharmaceutical, Sumitomo Pharma, Takeda Pharmaceutical, and Viatris Pharmaceutical within the past 3 years. TT and SS declare no conflict of interest. OT has received speaker's honoraria from Meiji‐Seika Pharma, Otsuka Pharmaceutical, Sumitomo Pharma, Viatris Pharmaceutical, and Takeda Pharmaceutical within the past 3 years. AI [Atsuko Inamoto] has received a research grant from MSD and speaker's honoraria from Daiichi Sankyo, Eisai, MSD, Otsuka Pharmaceutical, Sumitomo Pharma, Viatris Pharmaceutical, and Yoshitomi Yakuhin within the past 3 years. AI [Akira Iwanami] received research grants from Daiichi Sankyo, Eisai, Mochida Pharmaceutical, Otsuka Pharmaceutical, Shionogi Pharmaceutical, Sumitomo Pharma, and Takeda Pharmaceutical within the past 3 years.

## ETHICS STATEMENT

Approval of the Research Protocol by an Institutional Reviewer Board: The protocol for this research project has been approved by the Ethics Committee on Research Involving Human Subjects at Showa University (21‐048‐A). The protocols were performed according to the “Ethical Guidelines for Medical Research Involving Human Subjects” established by the Ministry of Health, Labour and Welfare and the Ministry of Education, Culture, Sports, Science and Technology in Japan.

Informed Consent: Written informed consent was obtained from all participants before administering the questionnaire, and information was extracted from their medical records. A clear explanation regarding the writing of the study and the fact that the participants could refuse to participate at any point was provided to each participant and notified on our website. Personal information was anonymized using numerical codes, and a designated information manager was appointed to oversee its management. The list of individuals and their corresponding anonymized numbers was stored on an in‐hospital server separated from the external network, and access was restricted using password settings.

Registry and the Registration No. of the Study/Trial: N/A.

Animal Studies: N/A.

Permission to reproduce material from other sources: N/A.

## Data Availability

Raw data were generated at Department of Psychiatry Showa University. Derived data supporting the findings of this study cannot be disclosed, for which patients' agreement was not acquired through the informed consent. Part of it will be available from the corresponding author on request.
